# Pediatric Intrafalcine Empyema from a Sinogenic Origin: A Case Report

**DOI:** 10.7759/cureus.1223

**Published:** 2017-05-04

**Authors:** Kyle Mueller, John Myseros

**Affiliations:** 1 Neurosurgery, Medstar Georgetown University Hospital; 2 Neurosurgery, Children's national medical center

**Keywords:** empyema, falx cerebri, venous congestion, antibiotics, surgical drainage, magnetic resonance imaging

## Abstract

Sinusitis and otitis are common within the pediatric population. If left untreated, these can extend intracranially and lead to the development of infections in the various intracranial compartments resulting in a high rate of morbidity and mortality. We report the first case of an intrafalcine empyema, absent subdural purulence, in a patient with the likely spread from a sinogenic origin. This case illustrates the novelty of this as a pathological entity as well as the surgical considerations for intrafalcine purulence in the absence of expected subdural collections.

## Introduction

Untreated sinusitis and otitis can lead to infections of the central nervous system (CNS) in children. Complications such as subdural empyema, epidural abscess, and intracranial abscess are well recognized and can have a high rate of morbidity and mortality if not diagnosed and treated promptly. We report a unique case presentation, findings and operative management of an intrafalcine empyema that extended from a sinogenic origin in a pediatric patient. The intrafalcine location for an empyema is novel, in that, all other reports of empyemas are located in other cranial compartments. Informed consent statement was obtained for this study.

## Case presentation

### History

A 10-year-old African-American male presented to our institution with one-week history of increasing headache along with pain and tenderness over his forehead. The headache had some initial improvement with Tylenol and Ibuprofen but continued to persist. He described his headache as being located frontally with a dull, throbbing sensation. He endorsed some subjective intermittent fever at the same time. There were no recent changes in his vision, trauma, rhinorrhea, seizures or sick contacts and the remainder of his review of systems was negative, with the exception of oral Montelukast for seasonal allergies.

### Physical exam and lab data

On presentation, his vital signs were the following: temperature: 40.1°C, heart rate: 130 bpm, blood pressure: 138/60 mmHg and 100% oxygen saturation on air. On examination, he was in no acute distress and had no neurological deficits. He had no nose drainage, and the middle of his forehead had some mild swelling that was soft. Laboratory studies showed a leukocytosis of 12.89 K/mcL with a reactive thrombocytosis of 452 K/mcL. The erythrocyte sedimentation rate (ESR) was 40 mm/hr and C-reactive protein (CRP) was 0.11 mg/dL.

### Imaging

**Figure 1:**
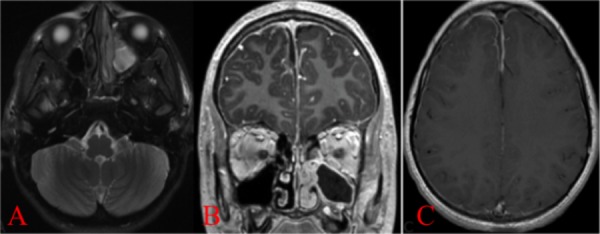
A) Axial T2 Magnetic resonance imaging (MRI) showing involvement of the paranasal sinuses. B) and C) axial and coronal gadolinium-enhanced MRI showing less than 2 mm subdural empyema, an extension from the sinusitis

A contrasted maxillofacial computed tomography (CT) was obtained which showed acute left-sided opacification in the left maxillary, ethmoid, and frontal sinuses as well as a subperiosteal abscess over the frontal bone. In addition, a gadolinium-enhanced brain magnetic resonance imaging (MRI)/ magnetic resonance venogram (MRV) was obtained. This is shown in figure 1 and demonstrates a left paranasal and midline purulence initially thought to be located in the subdural space. No area of venous infarction was noted.

### Initial management

**Figure 2:**
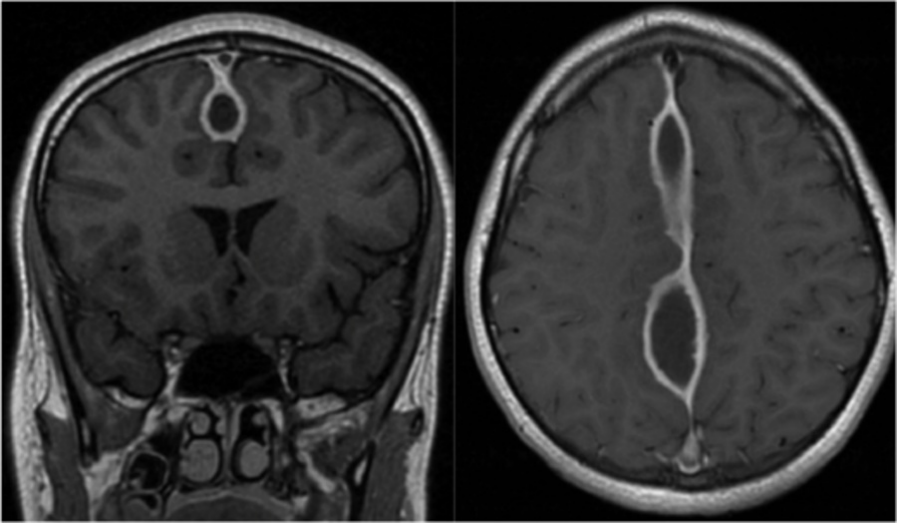
Interval MRI (one month later) showing enlargement and dural thickening of prior empyema

He was admitted to the hospital and started on broad-spectrum triple-antibiotic therapy which consisted of a combination of vancomycin, metronidazole, and ceftriaxone. He was taken to the operating room (OR) with otolaryngology ear, nose, and throat (ENT) for a functional endoscopic sinus surgery. No pathogen grew from the operative cultures. He was discharged home on continued intravenous (IV) antibiotics. He returned one month later with a routine, interval brain MRI shown in figure 2 demonstrating significant enlargement of the midline purulence along with dural thickening. During this time, he remained afebrile, asymptomatic, and with a resolution of his prior headache and forehead swelling. Given radiographic progression, despite aggressive antimicrobial therapy, it was decided to intervene surgically in order to potentially decrease the microbial burden and identify any undiagnosed disease. 

### Operative management

He was taken to the operating room and a right frontal craniotomy was performed exposing the sagittal sinus. The right interhemispheric fissure was accessed and no subdural purulence was noted. The right leaf of the falx was distended, fluctuant and was subsequently incised with purulent material expressed. This was cultured, evacuated and thoroughly irrigated with no defects or compromise of the contralateral left dural leaf noted.

### Postoperative course

**Figure 3:**
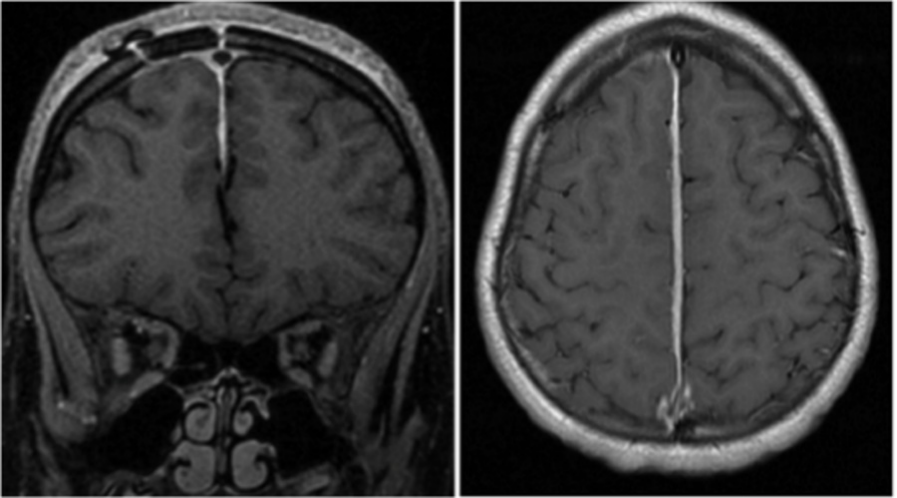
One-month post-operative (post-op) MRI showing complete resolution of prior intrafalcine empyema

His operative cultures did not grow any specific pathogens. The remainder of his hospital course was uneventful and he was subsequently discharged home on the same IV antibiotics as his initial admission. He was seen one month later in the outpatient clinic for his first postoperative appointment along with a postoperative MRI as shown in figure 3. This showed complete resolution of the prior intrafalcine empyema. He continued to have the resolution of his symptoms.

## Discussion

Sinusitis is the most common cause of extra-axial CNS infections in children in the Western world [1]. With the advent of modern antibiotics, CNS infections from sinogenic and otogenic sources have become less frequent. A review of the current medical literature did not reveal any report of an intracranial empyema located in the intrafalcine space. Reports of intracranial empyemas in other surgical compartments are well known.

CNS infections in children and adolescents can arise as extensions from both otologic and paranasal sinus disease. Left untreated infection may spread intracranially and lead to increased morbidity and mortality. Contiguous spread through erosion of the posterior wall of the frontal sinus is the most common route of spread. Most patients that develop subdural empyemas are male and between six and 20 years of age [1]. Other CNS infections that can be seen include cranial epidural abscesses, brain abscess and viral infections [2].

Historically, the prognosis for children with CNS infections was very poor. The advancement of antibiotics coupled with improvement in surgical asepsis has allowed these patients to have better outcomes. With sinusitis and otitis being common in the pediatric population, it is not unusual for infections to be present for days prior to diagnosis. Improved imaging modalities, such as MRI, have allowed for earlier and more precise evaluation of the extent of infectious involvement [2].

Keith reported the first surgical management of subdural empyema in 1949 [3]. Since that time, treatment has become more aggressive and combined medical and surgical therapies are implemented early in order to improve patient outcomes. Combined sinus drainage with ENT is often employed in order to reduce the overall infectious level. In select patients with small intracranial extension and no neurological deficits, observation with serial imaging may be indicated [4].

Our understanding of the pathological basis of the disease process has greatly improved over the years. Many large series in the literature, detail the relationship between sinusitis and otitis as being a primary source of infection that can extend intracranially if not treated [5-9]. Subdural empyemas, in particular, may lead to cortical venous thrombosis and potentially devastating venous congestion and infarction. [10] With regards to etiology, in our patient, with the absence of obvious subdural purulence, we speculate that a robust interfalcine venous system allowed for seeding, perhaps through transdural contamination.

## Conclusions

We report this case to add to the body of literature on CNS infections in children and adolescents. This case demonstrates the unique presentation of intracranial purulence within the dural leaflets of the falx cerebri, absent as a subdural collection. This is an unreported compartment for the development of an epyema. In these rare cases, where interhemispheric subdural empyema is common and expected, the absence of obvious disease upon initial exposure should prompt a consideration for opening and explore the intrafalcine space.
